# INFORMED CONSENT IN CLINICAL TRIALS FOR PERSONS WITH DEMENTIA

**DOI:** 10.1093/geroni/igz038.106

**Published:** 2019-11-08

**Authors:** Joan G Carpenter

**Affiliations:** University of Pennsylvania School of Nursing, Philadelphia, Pennsylvania, United States

## Abstract

Informed consent is one of the most important processes during the implementation of a clinical trial; special attention must be given to meeting the needs of persons with dementia in nursing homes who have impaired decision making capacity. We overcame several challenges during enrollment and consent of potential participants in a pilot clinical trial including: (1) the consent document was designed for legally authorized representatives however some potential participants were capable of making their own decisions; (2) the written document was lengthy yet all seven pages were required by the IRB; (3) the required legal wording was difficult to understand and deterred potential participants; and (4) the primary mode of communication was via phone. We tailored assent and informed consent procedures to persons with dementia and their legally authorized representative/surrogate decision maker to avoid risking an incomplete trial and to improve generalizability of trial results to all persons with dementia.

